# The new genus *Bambusiporia* and a new species of *Etheirodon* in *Steccherinaceae* (*Polyporales*, *Basidiomycota*) from China

**DOI:** 10.3897/mycokeys.130.183355

**Published:** 2026-03-20

**Authors:** Chao-Ge Wang, Xin Zhang, Zhan-Bo Liu, Jian Chen, Yi-Fei Sun, Yu-Cheng Dai, Ying-Da Wu

**Affiliations:** 1 State Key Laboratory of Efficient Production of Forest Resources, School of Ecology and Nature Conservation, Beijing Forestry University, Beijing 100083, China School of Ecology and Nature Conservation, Beijing Forestry University Beijing China; 2 Taohuayu Yellow River Floodplain Ecosystem Observation and Research Station of Henan Province, School of Life Sciences, Henan University, Zhengzhou 450046, China School of Life Sciences, Henan University Zhengzhou China; 3 College of Forestry, Sichuan Agricultural University, Chengdu 611130, China College of Forestry, Sichuan Agricultural University Chengdu China; 4 Forest Ecology and Conservation in the Upper Reaches of the Yangtze River Key Laboratory of Sichuan Province, Chengdu 611130, China Forest Ecology and Conservation in the Upper Reaches of the Yangtze River Key Laboratory of Sichuan Province Chengdu China; 5 Sichuan Mt. Emei Forest Ecosystem National Observation and Research Station, Leshan 614200, China Sichuan Mt. Emei Forest Ecosystem National Observation and Research Station Leshan China; 6 Key Laboratory of Forest and Grassland Fire Risk Prevention, Ministry of Emergency Management, China Fire and Rescue Institute, Beijing 102202, China Ministry of Emergency Management, China Fire and Rescue Institute Beijing China

**Keywords:** Divergence time estimation, phylogeny, polypore, taxonomy, wood-decaying fungi

## Abstract

*Steccherinaceae* is a speciose family of wood-inhabiting fungi containing various hymenophore configurations. Phylogenetic and morphological analyses of *Steccherinaceae* were carried out. Phylogenies were reconstructed using four genetic loci, including ITS, nLSU, *tef1*, and mtSSU. A new poroid genus, *Bambusiporia*, growing on dead bamboo from Yunnan Province in southwest China and typified by *Bambusiporia nivea*, is illustrated and described. It is characterized by resupinate basidiomata with a white pore surface when fresh, a monomitic hyphal system bearing clamp connections on generative hyphae, and ellipsoid, slightly thick-walled basidiospores. In addition, a new species in *Etheirodon*, *E. lilacinum*, is described, and it is characterized by resupinate to effused-reflexed basidiomata with a lilac hydnoid hymenophore when fresh; a dimitic hyphal system bearing clamp connections on generative hyphae; encrusted contextual generative hyphae; the absence of encrusted cystidia; and ellipsoid to broadly ellipsoid basidiospores measuring 4–4.5 × 3–3.8 µm. Moreover, the evolutionary timing of the main clades in *Steccherinaceae* was revealed based on conserved regions of two nuclear ribosomal genetic markers (ITS + nLSU). The ancestor of *Steccherinaceae* evolved during the early Cretaceous at 109.27 Myr [95% highest posterior density (HPD) of 81.91–141.87 Mya]. The initial diversification of the *Bambusiporia* clade occurred during the late Cretaceous with a mean crown age of 83.23 Myr [95% highest posterior density (HPD) of 56.74–112.09 Myr], earlier than other genera in *Steccherinaceae*. The *Etheirodon* clade emerged with a mean stem age of 51.98 Myr [95% highest posterior density (HPD) of 34.49–73.82 Myr] and a mean crown age of 19.56 Myr [95% highest posterior density (HPD) of 12.02–29.35 Myr].

## Introduction

*Polyporales (Basidiomycota)* is a major group of wood-inhabiting fungi, and extensive studies in the order have been carried out worldwide (Ryvarden 2015, 2016; Zhou et al. 2016; Ryvarden and Melo 2017; Cui et al. 2019; Ryvarden et al. 2022; Wu et al. 2022; Zhao et al. 2024). *Steccherinaceae* Parmasto is a family in *Polyporales*, typified by *Steccherinum* Gray, and was established by Parmasto (1968). Prior to the onset of the present study, the family included 23 genera, viz., *Antella* Miettinen, *Antrodiella* Ryvarden & I. Johans., *Atraporiella* Ryvarden, *Austeria* Miettinen, *Butyrea* Miettinen, *Cabalodontia* Piątek, *Caudicicola* Miettinen, M. Kulju & Kotir., *Citripora* Miettinen, *Etheirodon* Banker, *Flabellophora* G. Cunn., *Flaviporus* Murrill, *Frantisekia* Spirin & Zmitr., *Junghuhnia* Corda, *Lamelloporus* Ryvarden, *Loweomyces* (Kotl. & Pouzar) Jülich, *Metuloidea* G. Cunn., *Mycorrhaphium* Maas Geest., *Niemelaea* Zmitr., Ezhov & Khimich., *Nigroporus* Murrill, *Rhomboidia* C.L. Zhao, *Steccherinum*, *Trullella* Zmitr., and *Xanthoporus* Audet (Justo et al. 2017; Wu et al. 2022; Zhao et al. 2024). *Steccherinaceae* is characterized by resupinate, effused-reflexed to pileate or stipitate basidiomata with various hymenophores (such as smooth, granular, hydnoid, and poroid), a monomitic to dimitic hyphal system, generative hyphae with clamp connections or simple septa, allantoid, cylindrical, ellipsoid to subglobose basidiospores, and a white rot mode of wood decay (Maas Geesteranus 1971; Westphalen et al. 2021).

The three genera *Antrodiella*, *Junghuhnia*, and *Steccherinum* in *Steccherinaceae* are shown to be polyphyletic based on phylogenetic analyses (Miettinen et al. 2012; Yuan 2014; Westphalen et al. 2021; Yurchenko et al. 2023). Seven new genera have been segregated from these three genera during the last decade, namely *Antella*, *Austeria*, *Butyrea*, *Caudicicola*, *Citripora*, *Rhomboidia*, and *Trullella* (Miettinen and Ryvarden 2016; Kotiranta et al. 2017; Xu et al. 2020; Du et al. 2022).

The genus *Etheirodon* was established by Banker (1902). It has resupinate basidiomata with a fimbriate-rhizomorphic sterile margin, hydnoid to odontoid hymenophore, encrusted cystidia, and cylindrical to ellipsoid basidiospores. Prior to the onset of the present study, the genus contained three species, viz., *E. fimbriatus* (Pers.) Banker, *E. purpureus* Westphalen, and *E. roseoalbus* J.H. Dong & C.L. Zhao (Hjortstam and Ryvarden 2007; Westphalen et al. 2021; Dong et al. 2024). *Etheirodon* was treated as a synonym of *Odontia* Fr., a genus that belongs to *Thelephoraceae* in *Thelephorales* (Tedersoo et al. 2014).

In the present study, a phylogenetic assessment of *Steccherinaceae* based on four genetic markers (ITS + nLSU + *tef1* + mtSSU) was carried out. *Bambusiporia* gen. nov. with a new species, *B. nivea*, is proposed. Furthermore, a new species in *Etheirodon*, *E. lilacinum*, is described and illustrated. In addition, the main morphological characteristics of all 24 genera in *Steccherinaceae* are summarized. The molecular divergence times of *Steccherinaceae*, including *Bambusiporia* and *Etheirodon*, were analyzed based on the combined two-marker dataset (ITS + nLSU) in the present study.

## Materials and methods

### Morphological studies

The studied specimens are deposited in the Fungarium of the Institute of Microbiology, Beijing Forestry University (BJFC). Morphological descriptions are based on field notes and voucher specimens. The microscopic analysis follows Dai (2010) and Wu et al. (2022). Sections were studied at a magnification of up to 1000× using a Nikon Eclipse 80i microscope and phase contrast illumination. Descriptions of microscopic features and measurements were made from slide preparations stained with Cotton Blue and Melzer’s reagent. Basidiospores were measured from sections cut from the tubes. To represent the variation in the size of spores, 5% of measurements were excluded from each end of the range and are given in parentheses. In the description: **KOH** = 5% potassium hydroxide, **IKI** = Melzer’s reagent, **IKI–** = neither amyloid nor dextrinoid, **CB** = Cotton Blue, **CB–** = acyanophilous in Cotton Blue, **CB+** = cyanophilous in Cotton Blue, **L** = arithmetic average of spore length, **W** = arithmetic average of spore width, **Q** = L/W ratios, and ***n*** = number of basidiospores measured from the given number of specimens. Color terms follow Anonymous (1969) and Petersen (1996).

### DNA extraction, amplification, and sequencing

A CTAB rapid plant genome extraction kit-DN14 (Aidlab Biotechnologies Co., Ltd., Beijing) was used to obtain DNA from dried specimens, followed by the polymerase chain reaction (PCR) according to the manufacturer’s instructions with some modifications (Sun et al. 2020; Qin et al. 2025). The internal transcribed spacer regions (ITS), large subunit nuclear ribosomal RNA gene (nLSU), translation elongation factor 1-α gene (*tef1*), and mitochondrial small subunit rRNA gene (mtSSU) were amplified using the primer pairs ITS5/ITS4, LR0R/LR7, 985F/1567R, and MS1/MS2 (White et al. 1990; Hopple and Vilgalys 1999; Rehner and Buckley 2005) (https://sites.duke.edu/vilgalyslab/rdna_primers_for_fungi/).

The PCR procedure for ITS, *tef1*, and mtSSU was as follows: initial denaturation at 95 °C for 3 min, followed by 34 cycles at 94 °C for 40 s, annealing at 54 °C for ITS and 56 °C for *tef1* and mtSSU for 45 s, and extension at 72 °C for 1 min, with a final extension at 72 °C for 10 min. The PCR procedure for nLSU was as follows: initial denaturation at 94 °C for 1 min, followed by 34 cycles of denaturation at 94 °C for 30 s, annealing at 50 °C for 1 min, and extension at 72 °C for 1.5 min, with a final extension at 72 °C for 10 min. The PCR products were purified and sequenced at the Beijing Genomics Institute (BGI), China, with the same primers as used in PCR. Newly generated sequences were deposited in GenBank. All sequences analyzed in this study are listed in Table 1.

**Table 1.** A list of species, specimens, and GenBank accession numbers of sequences used in this study.

Species name	Sample no.	Location	GenBank accession no.			
			ITS	nLSU	mtSSU	*tef1*
*Agaricus campestris*	LAPAG370	—	KM657927	KR006607	—	—
*Amylocorticium cebennense*	CFMR: HHB-2808	USA	GU187505	GU187561	—	—
*Antella chinensis*	Dai 9019 (holotype)	China	JX110844	KC485542	—	—
*Antella niemelaei*	Renvall 3218	Finland	AF126876	—	—	—
*Antrodiella foliaceodentata*	X 1238	Russia	JN710515	JN710515	JN710659	—
*Antrodiella semisupina*	X 242	Canada	JN710521	JN710521	—	—
*Antrodiella* sp.	X 418	Japan	JN710523	JN710523	—	—
*Aphanobasidium pseudotsugae*	CFMR: HHB-822	USA	GU187509	GU187567	—	—
*Athelia epiphylla*	CFMR: FP-100564	USA	GU187501	GU187558	—	—
*Atraporiella neotropica*	Ryvarden 44447 (holotype)	Belize	HQ659221	HQ659221	—	—
*Atraporiella yunnanensis*	CLZhao 605 (holotype)	China	MF962483	MF962486	—	—
*Austeria citrea*	X 1171	New Zealand	JN710511	—	—	—
*Austeria citrea*	PDD 96654	New Zealand	MK404662	—	—	—
***Bambusiporia nivea***	Dai 22451 (holotype)	China	**PP907129**	**PP907121**	—	—
***Bambusiporia nivea***	Dai 22477	China	**PP907130**	**PP907122**	—	—
*Bjerkandera adusta*	Dai 14516	China	MW507097	MW520204	—	—
*Boletopsis leucomelaena*	AFTOL-ID 1527	USA	DQ484064	DQ154112	—	—
*Boletus edulis*	HMJAU4637	—	JN563894	KF112455	—	—
*Bondarzewia tibetica*	Yu 56	China	KT693203	KT693205	—	—
*Butyrea japonica*	Li 1648	China	KC485536	KC485553	—	—
*Butyrea luteoalba*	isolate 5403	Estonia	JN710558	JN710558	JN710682	JN710719
*Cabalodontia delicata*	MV 370	—	MT849298	—	—	—
*Cabalodontia delicata*	SP 512584 (holotype)	Brazil	NR174056	—	—	—
*Cabalodontia queletii*	CBS 233.56	France	MH857599	MH869147	—	—
*Callistosporium graminicolor*	AFTOL-ID 978	USA	DQ484065	AY745702	—	—
*Ceriporia allantospora*	RLG-10478 (holotype)	USA	KP135039	—	—	—
*Ceriporia aurantiocarnescens*	Dai 17951	China	MW491774	MW491764	—	—
*Ceriporia crassa*	Dai 22034 (holotype)	China	OQ476823	OQ476769	—	—
*Ceriporia griseoviolascens*	Dai 13202	France	OQ476825	OQ476771	—	—
*Ceriporia hinnulea*	Cui 11291 (holotype)	China	OQ476826	OQ476772	—	—
*Ceriporia mellita*	BR 4865	France	KX236485	KX236485	—	—
*Ceriporia sinoviridans*	Dai 13621A (holotype)	China	MW491781	MW491771	—	—
*Ceriporia spissa*	Dai 19164	Canada	OQ476845	OQ476789	—	—
*Ceriporia subviridans*	Cui 8012 (holotype)	China	KC182774	—	—	—
*Ceriporia viridans*	Dai 17003	China	OQ476847	OQ476790	—	—
*Citripora afrocitrina*	X 525	Uganda	JN710507	JN710507	JN710655	JN710710
*Citripora bannaensis*	X 243	China	JN710526	JN710526	—	—
*Cotylidia* sp.	MB5	—	AY854079	AY629317	—	—
*Crystallicutis* sp.	Dai 6090	China	JX623934	JX644066	—	—
*Crystallicutis serpens*	HHB-15692-Sp	USA	KP135031	KP135200	—	—
*Cymatoderma* sp.	OMC 1427	USA	KY948826	KY948872	—	—
*Etheirodon aff. fimbriatus*	HHB-2878-sp	USA	KY948822	KY948864	—	—
*Etheirodon cf. fimbriatus*	KUC 20121109-29	Korea	KJ668456	KJ668307	—	—
*Etheirodon cf. fimbriatus*	Dai 24450	China	PP907136	PP907128	—	
*Etheirodon cf. fimbriatus*	CLZhao 13977	China			—	—
*Etheirodon fimbriatus*	KHL 11905	Sweden	JN710530	JN710530	JN710667	—
*Etheirodon fimbriatus*	HR 97926	—	MT849299	—	—	MT833937
*Etheirodon fimbriatus*	HR 98811	—	MT849300	—	—	MT833938
***Etheirodon lilacinum***	Dai 23571 (holotype)	China	**PP907131**	**PP907123**	—	—
***Etheirodon lilacinum***	Dai 23574	China	**PP907132**	**PP907124**	**PX649048**	**PX667841**
***Etheirodon lilacinum***	Dai 23131	China	**PP907133**	**PP907125**	**PX649047**	**PX667842**
***Etheirodon lilacinum***	Dai 23568	China	**PP907134**	**PP907126**	**PX649046**	**PX667843**
***Etheirodon lilacinum***	Dai 23140	China	**PP907135**	**PP907127**	—	—
*Etheirodon purpureus*	MCW 642/18 (holotype)	Brazil	MT849301	MT849301	—	MT833939
*Etheirodon roseoalbus*	CLZhao 24770 (holotype)	China	OR096187	OR461452	—	—
*Etheirodon roseoalbus*	CLZhao 24903	China	OR096188	OR461453	—	—
*Flabellophora* sp.1	X 1357	Indonesia	JN710533	JN710533	—	—
*Flabellophora* sp.3	X 1277	Indonesia	JN710535	JN710535	JN710669	—
*Flaviporus albus*	GXU 5765 (holotype)	China	OQ981991	OQ981993	—	—
*Flaviporus brownii*	X 1216	Ecuador	JN710537	JN710537	—	—
*Flaviporus subundatus*	MCW 457/13	—	KY175005	—	—	—
*Flaviporus tenuis*	MCW 44213	—	KY175001	KY175001	—	—
*Frantisekia mentschulensis*	AH 1377	Austria	JN710544	JN710544	—	—
*Frantisekia mentschulensis*	BRNM 710170	Czechia	FJ496670	FJ496728	FJ496748	—
*Frantisekia ussurii*	Wei 3081	China	KC485527	KC485545	—	—
*Frantisekia ussurii*	Dai 8249	China	KC485526	—	—	—
*Gloeophyllum sepiarium*	Wilcox-3BB	USA	HM536091	HM536061	—	—
*Gloeoporus dichrous*	Dai 23260	China	OQ476852	OQ476795	—	—
*Gloeoporus pannocinctus*	FP 135015	USA	MG572755	MG572739	—	—
*Hydnochaete duportii*	AFTOL-ID 666	—	DQ404386	AY635770	—	—
*Hydnophanerochaete odontoidea*	CWN 00776	China	LC363487	GQ470663	—	—
*Hyphoderma litschaueri*	FP-101740-Sp	USA	KP135295	KP135219	—	—
*Hyphoderma mutatum*	HHB-15479-Sp	USA	KP135296	KP135221	—	—
*Hyphoderma praetermissum*	AFTOL-ID 518	—	AY854081	AY700185	—	—
*Hyphoderma setigerum*	FD-312	USA	KP135297	KP135222	—	—
*Hypochnicium karstenii*	NH 10924	Sweden	DQ677510	DQ677510	—	—
*Hypochnicium polonense*	NH 12117	Russia	EU118635	EU118635	—	—
*Jaapia argillacea*	CBS: 252.74	Netherlands	GU187524	GU187581	—	—
*Junghuhnia crustacea*	X 262	Indonesia	JN710553	JN710553	JN710678	—
*Junghuhnia crustacea*	X 1127	Indonesia	JN710554	JN710554	—	—
*Junghuhnia micropora*	Spirin 2652	Russia	JN710559	JN710559	JN710683	JN710720
*Lactarius deceptivus*	AFTOL-ID 682	USA	AY854089	AY631899	—	—
*Lamelloporus americanus*	X 670	Ecuador	JN710567	JN710567	—	—
*Lamelloporus americanus*	RLC 779	Ecuador	OQ871855	—	—	—
*Leptosporomyces raunkiaeri*	CFMR: HHB-7628	USA	GU187528	GU187588	—	—
*Loweomyces fractipes*	X 1253	USA	JN710569	JN710569	JN710689	—
*Loweomyces fractipes*	X 1250	USA	JN710568	JN710568	—	—
*Loweomyces wynneae*	X 1215	Denmark	JN710604	JN710604	JN710709	—
*Luteochaete subglobosa*	GC 1605-4	China	MZ636995	MZ637156	—	—
*Meripilus albostygius*	Kout 1807/15.1 (holotype)	Puerto Rico	OM669892	OM669976	—	—
*Meripilus crataegi*	Dai 15497 (holotype)	China	KY131845	KY131904	—	—
*Meripilus eminens*	Dai 22472	China	OM669900	OM669983	—	—
*Meripilus expallescens*	Dai 21060	Belarus	MT840130	MT840148	—	—
*Meripilus furcatus*	TAA 150972 (holotype)	Russia	KY131853	KY131910	—	—
*Meripilus giganteus*	Cui 9202	UK	OM669888	OM669973	—	—
*Meripilus neovitreus*	JV 1009/59 (holotype)	USA	OM669908	OM669990	—	—
*Meripilus pouzarii*	Dai 21043	Belarus	MT840124	MT840142	—	—
*Meripilus sanguinolentus*	JV 1310/11	Czechia	OM669920	OM669998	—	—
*Meripilus srilankensis*	Dai 19535 (holotype)	Sri Lanka	OM669924	OM670001	—	—
*Meripilus sumstinei*	Russell 5913	USA	MN906088	—	—	—
*Meripilus tibeticus*	Cui 9588	China	KY131873	KY131929	—	—
*Meripilus vitreus*	Dai 12685	Czechia	MT840115	MT840133	—	—
*Meruliopsis albomellea*	Dai 15205 (holotype)	China	KX494574	KX494578	—	—
*Meruliopsis bambusicola*	Dai 21944 (holotype)	China	OQ476864	OQ476806	—	—
*Meruliopsis crassitunicata*	Dai 10833	China	JX623935	JX644064	—	—
*Meruliopsis nanlingensis*	Dai 13414	China	OQ476868	OQ476809	—	—
*Meruliopsis tarda*	Dai 10226	China	JX623945	—	—	—
*Meruliopsis taxicola*	Dai 22625	China	OL457966	OL457436	—	—
*Metuloidea murashkinskyi*	X 449	Russia	JN710588	JN710588	—	—
*Metuloidea rhinocephala*	X 460	Australia	JN710562	JN710562	JN710686	—
*Mycorrhaphium adustum*	X 8024	USA	JN710573	JN710573	JN710692	—
*Mycorrhaphium subadustum*	Yuan 12976 (holotype)	China	MW491378	MW488040	—	—
*Neolentinus adhaerens*	DAOM 214911	—	HM536096	HM536071	—	—
*Niemelaea balaenae*	H 7002389	Canada	FJ496669	FJ496717	FJ496746	—
*Niemelaea consobrina*	Rivoire 977	France	FJ496667	FJ496716	—	—
*Nigroporus stipitatus*	X 546	Cameroon	JN710574	JN710574	—	—
*Nigroporus vinosus*	X 839	USA	JN710575	JN710575	—	—
*Panus fragilis*	HHB-11042-Sp	USA	KP135328	KP135233	—	—
*Phanerochaete alnea*	FP-151125	USA	KP135177	MZ637181	—	—
*Phanerochaetella exilis*	HHB-6988	USA	KP135001	KP135236	—	—
*Phanerochaetella xerophila*	HHB-8509-Sp	USA	KP134996	KP135259	—	—
*Phlebiopsis gigantea*	FCUG 1417	Norway	MZ637051	AF141634	—	—
*Phlebiporia bubalina*	Dai 13168	China	KC782526	KC782528	—	—
*Podoscypha multizonata*	Jahn 751012	Germany	EU118663	EU118663	—	—
*Podoscypha venustula*	LR 40821	Venezuela	JX109851	JX109851	—	—
*Podoserpula ailaoshanensis*	ZJL2015015	China	KU324484	KU324487	—	—
*Pseudolagarobasidium baiyunshanense*	Han 405 (holotype)	China	MT428549	MT428547	—	—
*Radulodon americanus*	CFMR-HHB 11240	USA	JQ070174	—	—	—
*Radulodon yunnanensis*	Cui 17979 (holotype)	China	OM971917	OM971898	—	—
*Resiniporus pseudogilvescens*	Wu 1209-46	China	KY688203	MZ637268	—	—
*Rhomboidia wuliangshanensis*	CLZhao 4406 (holotype)	China	MK860715	MK860710	—	—
*Rhomboidia wuliangshanensis*	CLZhao 4411	China	MK860716	MK860711	—	—
*Russula emeticicolor*	FH12253	Germany	KT934011	KT933872	—	—
*Schizophyllum radiatum*	AFTOL-ID-516	Panama	AY571060	AY571023	—	—
*Scopuloides hydnoides*	FP-150473	USA	KP135355	KP135284	—	—
*Serpula himantioides*	MUCL: 30528	Belgium	GU187545	GU187600	—	—
*Skeletocutis novae-zelandiae*	Ryvarden 38641	New Zealand	JN710582	JN710582	—	—
*Spongipellis quercicola*	Cui 10009 (holotype)	China	OM971919	OM971899	—	—
*Spongipellis sibirica*	Dai 1723	China	OM971921	—	—	—
*Spongipellis spumeus*	BRNM 734877	Czechia	HQ728283	HQ729018	—	—
*Spongiporus leucospongia*	OKM-4335	USA	KC585395	KC585228	—	—
*Steccherinum autumnale*	Spirin 2957	Russia	JN710549	JN710549	JN710675	JN710716
*Steccherinum fragile*	Dai 19972	China	MW364629	MW364627	—	—
*Steccherinum fragile*	Dai 20479 (holotype)	China	MW364628	MW364626	—	—
*Steccherinum incrustans*	Dai 19442	China	ON182084	ON182087	—	—
*Steccherinum incrustans*	X 1345	China	JN710550	JN710550	—	—
*Steccherinum juniperi*	Dai 23930	China	OP956076	—	—	—
*Steccherinum meridionale*	CBS 125887	New Zealand	MH864086	MH875544	—	—
*Steccherinum nandinae*	Dai 21108	China	MN833678	MN833680	—	—
*Steccherinum nitidum*	KHL 11903	Sweden	JN710560	JN710560	JN710684	JN710721
*Steccherinum ochraceum*	KHL 11902	Sweden	JN710590	JN710590	—	—
*Steccherinum polycystidiferum*	RP 140	—	KY174996	KY174996	—	—
*Steccherinum subcollabens*	Dai 19345 (holotype)	China	MN871759	MN877772	—	—
*Terana caerulea*	FP-104073	USA	KP134980	KP135276	—	—
*Tomentella* sp.	AFTOL-ID 1016	USA	DQ835998	DQ835997	—	—
*Trullella dentipora*	X 200	Venezuela	JN710512	JN710512	—	—
*Trullella polyporoides*	X 510	Venezuela	JN710602	JN710602	—	—
*Xanthoporus syringae*	Gothenburg 1488	Sweden	JN710607	JN710607	—	—
*Xanthoporus syringae*	X 339	Finland	JN710606	JN710606	—	—

New taxa and newly generated sequences are in bold.

### Sequence alignment

Sequences generated from this study were aligned with additional sequences downloaded from GenBank using BioEdit (Hall 1999). The final ITS, nLSU, *tef1*, and mtSSU datasets were subsequently aligned using MAFFT v.7 under the E-INS-i strategy (command line: mafft –genafpair –maxiterate 1000) (Katoh and Standley 2013) and visualized in BioEdit. Alignments were manually concatenated and processed further in Mesquite v.3.2 (Maddison and Maddison 2017).

### Phylogenetic analyses

In this study, one combined matrix was reconstructed for phylogenetic analyses; a four-marker dataset (ITS + nLSU + *tef1* + mtSSU) was used to determine the phylogenetic position of the new species. The sequence alignments and the retrieved topologies were deposited in TreeBase (http://www.treebase.org), under accession ID: 32532 (Reviewer access URL: http://purl.org/phylo/treebase/phylows/study/TB2:S32532?x-access-code=74c447e7541163ef3f4cefaf104f2b6&format=html). Sequences of *Hyphoderma setigerum* (Fr.) Donk and *Hyphoderma litschaueri* (Burt) J. Erikss. & Å. Strid, obtained from GenBank, were used as the outgroups (Justo et al. 2017). The phylogenetic analyses followed the approach of Han et al. (2016) and Zhu et al. (2019). Maximum likelihood (ML) and Bayesian inference (BI) analyses were performed based on the four-marker dataset. The combined dataset (ITS1 + 5.8S + ITS2 + nLSU + *tef1* + mtSSU) was partitioned into six subsets, and the best-fit substitution model for each partition was selected under the Akaike Information Criterion (AIC) using PartitionFinder within PhyloSuite v1.2.3 (Lanfear et al. 2017; Zhang et al. 2020). Then, the models were estimated separately for ITS1, 5.8S, ITS2, nLSU, *tef1*, and mtSSU, and Bayesian inference (BI) was analyzed using a partitioned, mixed-model run.

Sequences were analyzed using maximum likelihood (ML) with RAxML-HPC v.8.2.12 through the CIPRES Science Gateway (Miller et al. 2010). Branch support (BT) for ML analysis was determined by 1000 bootstrap replicates. Bayesian phylogenetic inference and Bayesian posterior probabilities (BPP) were computed with MrBayes 3.2.6. Four Markov chains were run for 3 million generations until the split deviation frequency value was less than 0.01, and trees were sampled every 100 generations. The first 25% of the sampled trees were discarded as burn-in, and the remaining ones were used to infer a majority rule consensus and calculate Bayesian posterior probabilities (BPP) of the clades. All trees were viewed in FigTree v.1.4.3 (http://tree.bio.ed.ac.uk/software/figtree/). Branches that received bootstrap support ML ≥ 75% and BPP ≥ 0.95 were considered significantly supported. The ML bootstrap supports of ≥ 50% and BPP of ≥ 0.90 are presented on the topology from the ML analysis.

### Divergence time estimation

*Archaeomarasmius leggetti* Hibbett et al. and *Quatsinoporites cranhamii* Smith et al. were selected as fossil calibrations in the divergence times of *Steccherinaceae*, including *Etheirodon* and the new genus *Bambusiporia*. *A. leggetti* was recorded at 94–90 Myr (Hibbett et al. 1997) as the representative of the minimum age of *Tricholomataceae* R. Heim ex Pouzar belonging to the *Agaricales*. *Q. cranhamii*, found in marine calcareous concretions on Vancouver Island, was considered to represent the minimum divergence time of the *Hymenochaetales* at 113 Myr (Smith et al. 2004). Divergence times were estimated using BEAST v2.6.5 (Bouckaert et al. 2014) based on a dataset of ITS + nLSU. The GTR + G + I substitution model was selected as the best-fit model for the two-marker dataset using MrModelTest2-v.2.4 (Nylander 2004). An XML file was executed using BEAUti v2. The clock model was set to an uncorrelated lognormal relaxed clock (Drummond et al. 2006; Lepage et al. 2007). The Yule process speciation was used as the tree prior (Gernhard 2008). For calibration, a gamma distribution prior (scale = 20, shape = 1) was specified for the *Agaricales* (offset = 90 Myr) and *Hymenochaetales* (offset = 125 Myr) clades (Sánchez-Ramírez et al. 2014; Zhao et al. 2016, 2017). All the ucld.mean parameters for different genes were set to uniform. Monte Carlo Markov chains were run for 100 million generations, logging states every 1000 generations. The resulting log file was checked for convergence of the chains using Tracer v1.6 (Rambaut et al. 2013; http://tree.bio.ed.ac.uk/software/tracer/). An ultrametric maximum clade credibility (MCC) tree was summarized using TreeAnnotator v2.6.5, discarding 20% of states as burn-in and annotating clades with ≥ 0.8 posterior probability. FigTree v1.4.3 (http://tree.bio.ed.ac.uk/software/figtree/) was used to visualize the resulting tree and to obtain the means and 95% HPD (Drummond and Rambaut 2007). A 95% HPD marks the shortest interval that contains 95% of the values sampled.

## Results

### Molecular phylogeny

**Figure 1.**
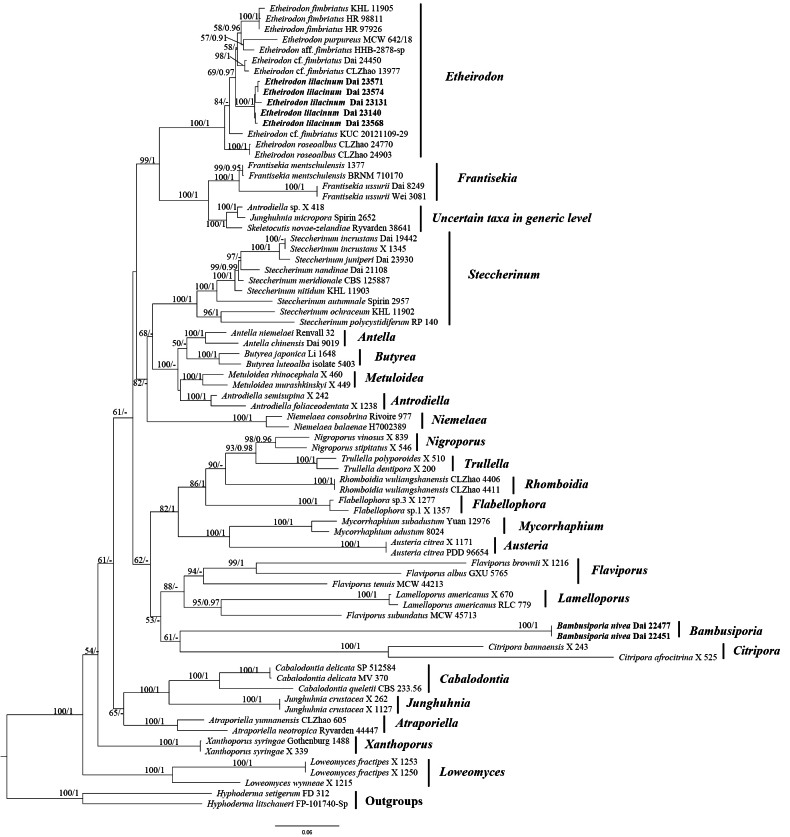
ML analysis of *Steccherinaceae* based on a dataset of ITS + nLSU + *tef1* + mtSSU. ML bootstrap values higher than 50% and Bayesian posterior probability values more than 0.90 are shown. New taxa are in bold.

The combined four-marker dataset (ITS + nLSU + *tef1* + mtSSU) included sequences from 77 samples representing 55 taxa, and the dataset had an aligned length of 3397 characters. The phylogenetic reconstruction performed with maximum likelihood (ML) and Bayesian inference (BI) analyses on the combined dataset showed a similar topology and only minor differences in statistical support. The substitution model employed for the ML analysis was GTRGAMMA, and all sequences divided into six partitions were Subset1 (ITS1) = 1–335, Subset2 (5.8S) = 336–510, Subset3 (ITS2) = 511–882, Subset4 (nLSU) = 883–2256, Subset5 (*tef1*) = 2257–2814, and Subset6 (mtSSU) = 2815–3397. The best model fit applied in the Bayesian analysis for each region of the six partitions was ITS1 (GTR+I+G), 5.8S (TVMEF+G), ITS2 (TVM+I+G), nLSU (GTR+I+G), *tef1* (TRNEF+G), and mtSSU (GTR+I+G); lset nst for ITS1, 5.8S, ITS2, nLSU, *tef1*, and mtSSU = 6; rates = invgamma (ITS1, ITS2, nLSU, and mtSSU); gamma (5.8S, *tef1*); and prset statefreqpr = dirichlet (1, 1, 1, 1). Bayesian analysis resulted in a nearly congruent topology with an average standard deviation of split frequencies = 0.004359, and thus only the ML tree is provided (Fig. 1). The phylogeny (Fig. 1) indicated the taxonomic relationship of genera in *Steccherinaceae*, and the *Bambusiporia* clade is phylogenetically close to the *Citripora* clade without strong support. The new species *Etheirodon lilacinum* differed from the other three species, *E. fimbriatus*, *E. purpureus*, and *E. roseoalbus*, nested in the *Etheirodon* clade. The polypore genera *Antrodiella*, *Junghuhnia*, and *Skeletocutis* are polyphyletic within the *Steccherinaceae* and *Incrustoporiaceae*, respectively. Three specimens (X418, Spirin 2652, and Ryvarden 38641), identified as *Antrodiella* sp., *Junghuhnia micropora*, and *Skeletocutis novae-zelandiae*, formed an independent clade in the previous study (Miettinen et al. 2012). In our phylogenetic tree, these three specimens are still assigned the original taxon names in accordance with the taxonomic concept of Miettinen et al. (2012).

### Divergence time estimation

**Figure 2.**
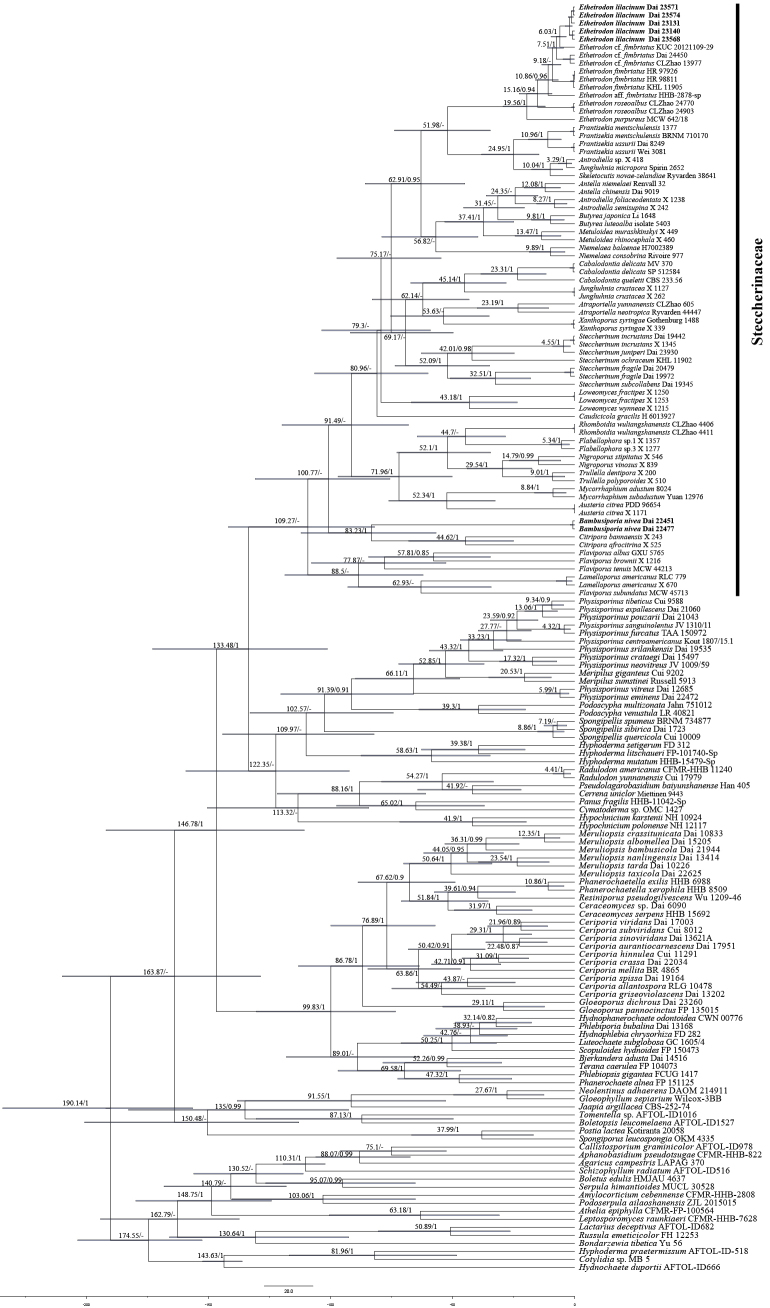
Divergence time estimation of *Steccherinaceae* from Bayesian evolutionary analysis sampling tree based on the conserved regions of two DNA fragments (ITS + nLSU). Posterior probabilities are not less than 0.80, and the mean ages (Myr) of each node are annotated. The 95% highest posterior densities of divergence time estimation are marked by horizontal bars.

The MCMC tree (Fig. 2) shows that the ancestor of the *Steccherinaceae* evolved during the early Cretaceous at 109.27 Myr [95% highest posterior density (HPD) of 81.91–141.87 Myr]. The initial diversification of the *Etheirodon* clade occurred during the Eocene in the Paleogene Period with a mean stem age of 51.98 Myr [95% highest posterior density (HPD) of 34.49–73.82 Myr] and a mean crown age of 19.56 Myr [95% highest posterior density (HPD) of 12.02–29.35 Myr]. The new genus *Bambusiporia* was estimated at 83.23 Myr, emerging in the late Cretaceous, and the posterior probability is up to 1. However, the divergence time of the *Citripora* clade emerged with a mean crown age of 44.62 Myr [95% highest posterior density (HPD) of 25–67.98 Myr], which belongs to the Eocene of the Paleogene Period. The international chronostratigraphic chart follows Cohen et al. (2013; updated) (URL: https://stratigraphy.org/chart/).

### Taxonomy

#### Bambusiporia

Taxon classificationFungiPolyporalesSteccherinaceae

Y.C. Dai, Xin Zhang & Chao G. Wang gen. nov.

F70CD51E-EE21-5787-934F-682D922986C0

MB854326

##### Etymology.

*Bambusiporia* (Lat.): refers to the genus having resupinate basidiomata and growing on bamboo.

##### Type species.

*Bambusiporia nivea* Y.C. Dai & Chao G. Wang, sp. nov.

##### Description.

Basidiomata tiny, annual, resupinate, detachable, soft when fresh. Poroid hymenophore, white when fresh. Hyphal system monomitic; generative hyphae bearing clamp connections, hyaline, thin- to slightly thick-walled. Cystidia and cystidioles absent. Basidiospores ellipsoid, hyaline, slightly thick-walled, smooth, sometimes with one or two small guttules, IKI−, weakly CB+.

#### Bambusiporia nivea

Taxon classificationFungiPolyporalesSteccherinaceae

Y.C. Dai, Xin Zhang & Chao G. Wang sp. nov.

6C1CCB70-933A-5088-A1F9-810BB54B45E0

MB854327

Figs 3, 4

##### Holotype.

China • Yunnan Province, Zhaotong, Huanglianhe Forest Park, on dead bamboo, 30 June 2021, Dai 22451 (BJFC037035).

**Figure 3.**
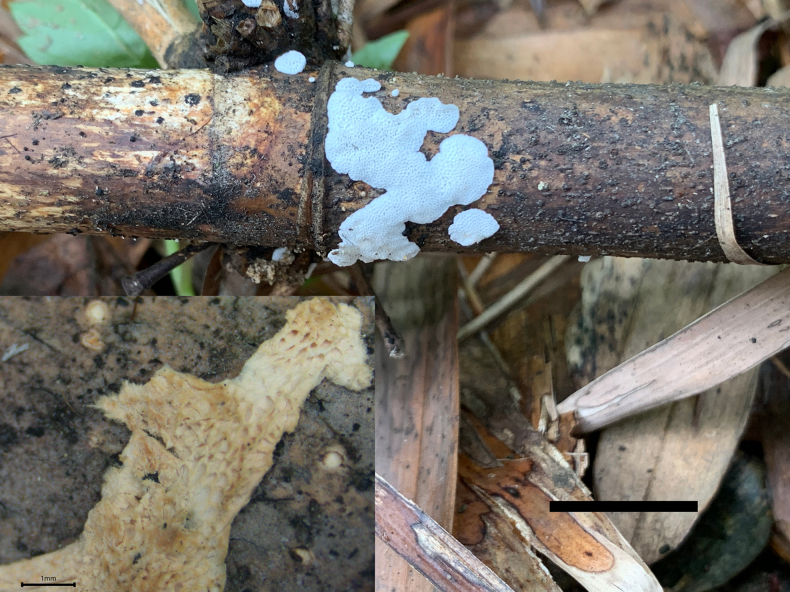
Basidiomata of *Bambusiporia nivea* (holotype, Dai 22451). Scale bar: 1 cm.

**Figure 4.**
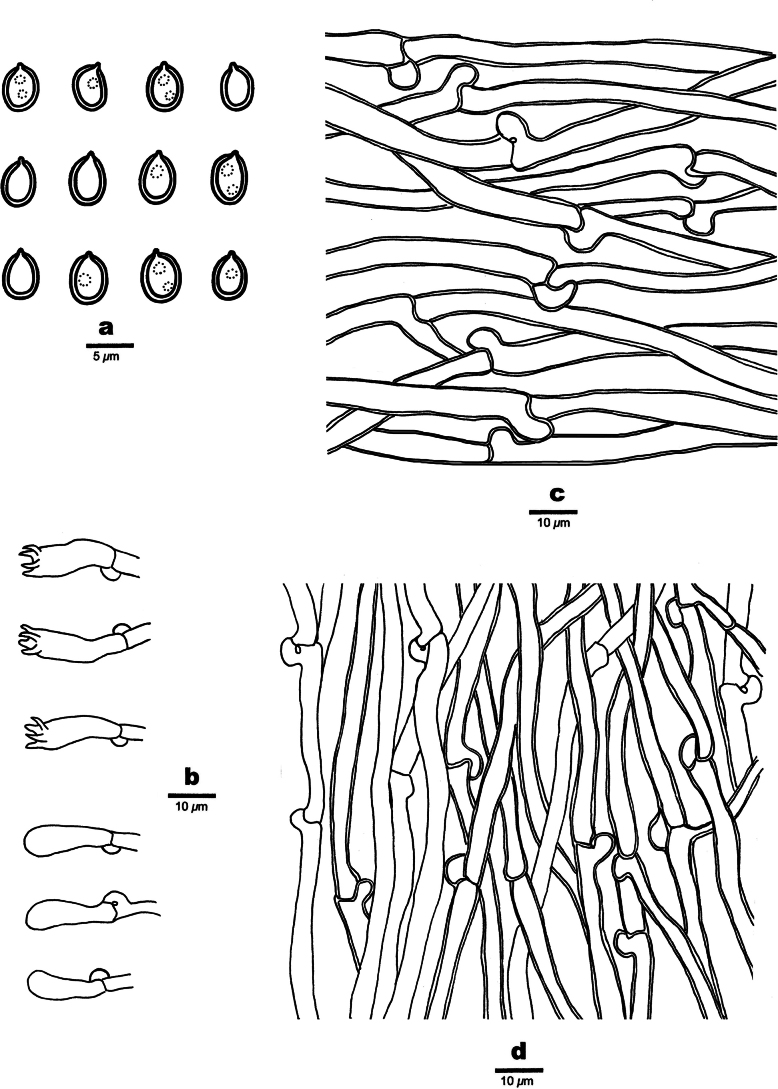
Microscopic structures of *Bambusiporia nivea* (holotype, Dai 22451). **a**. Basidiospores; **b**. Basidia and basidioles; **c**. Hyphae from subiculum; **d**. Hyphae from trama.

##### Etymology.

*Nivea* (Lat.): refers to the species having white pore surface when fresh.

##### Description.

***Basidiomata***. Annual, resupinate, separate, soft, without odor or taste when fresh, soft corky when dry, up to 1 cm long, 0.8 cm wide, 2 mm thick at center. Pore surface white when fresh, becoming pinkish buff upon drying; sterile margin very narrow to almost absent; pores round to angular, 4–6 per mm; dissepiments thin, entire. Subiculum thin, cream, corky, up to 0.5 mm thick. Tubes concolorous with pore surface, soft corky, up to 1.5 mm long.

***Hyphal structure***. Hyphal system monomitic; generative hyphae bearing clamp connections, hyaline, IKI−, CB−; tissues unchanged in KOH.

***Subiculum***. Generative hyphae slightly thick-walled with a wide lumen, occasionally branched, more or less flexuous, loosely interwoven, 4–7 μm in diam. Irregular crystals present amongst subicular hyphae.

***Tubes***. Generative hyphae thin- to slightly thick-walled with a wide lumen, occasionally branched, more or less flexuous, 3–5 μm in diam. Cystidia and cystidioles absent. Basidia clavate, with four sterigmata and a basal clamp connection, 15–20 × 6–6.5 μm; basidioles in shape similar to basidia but smaller.

***Spores***. Basidiospores ellipsoid, hyaline, slightly thick-walled, smooth, sometimes with one or two small guttules, IKI−, weakly CB+, (3.8–)4–4.6(–4.8) × 3–3.7(–3.8) µm, L = 4.19 μm, W = 3.3 μm, Q = 1.26–1.28 (n = 60/2).

***Type of rot***. White rot.

##### Additional specimen examined.

China • Yunnan Province, Zhaotong, Huanglianhe Forest Park, on dead bamboo, 30 June 2021, Dai 22477 (BJFC037061).

##### Notes.

*Bambusiporia nivea* is characterized by resupinate basidiomata with a white pore surface when fresh, round to angular pores of 4–6 per mm, a monomitic hyphal system with generative hyphae bearing clamp connections, ellipsoid, slightly thick-walled basidiospores measuring 4–4.6 × 3–3.7 µm, and growing on dead bamboo in southwest China.

#### Etheirodon lilacinum

Taxon classificationFungiPolyporalesSteccherinaceae

Y.C. Dai & Chao G. Wang sp. nov.

D0633E10-FB1B-542D-9241-126CD8067065

MB854328

Figs 5, 6

##### Holotype.

China • Xizang Autonomous Region, Linzhi, Bomi County, on fallen trunk of *Betula*, 26 October 2021, Dai 23568 (BJFC038140).

**Figure 5.**
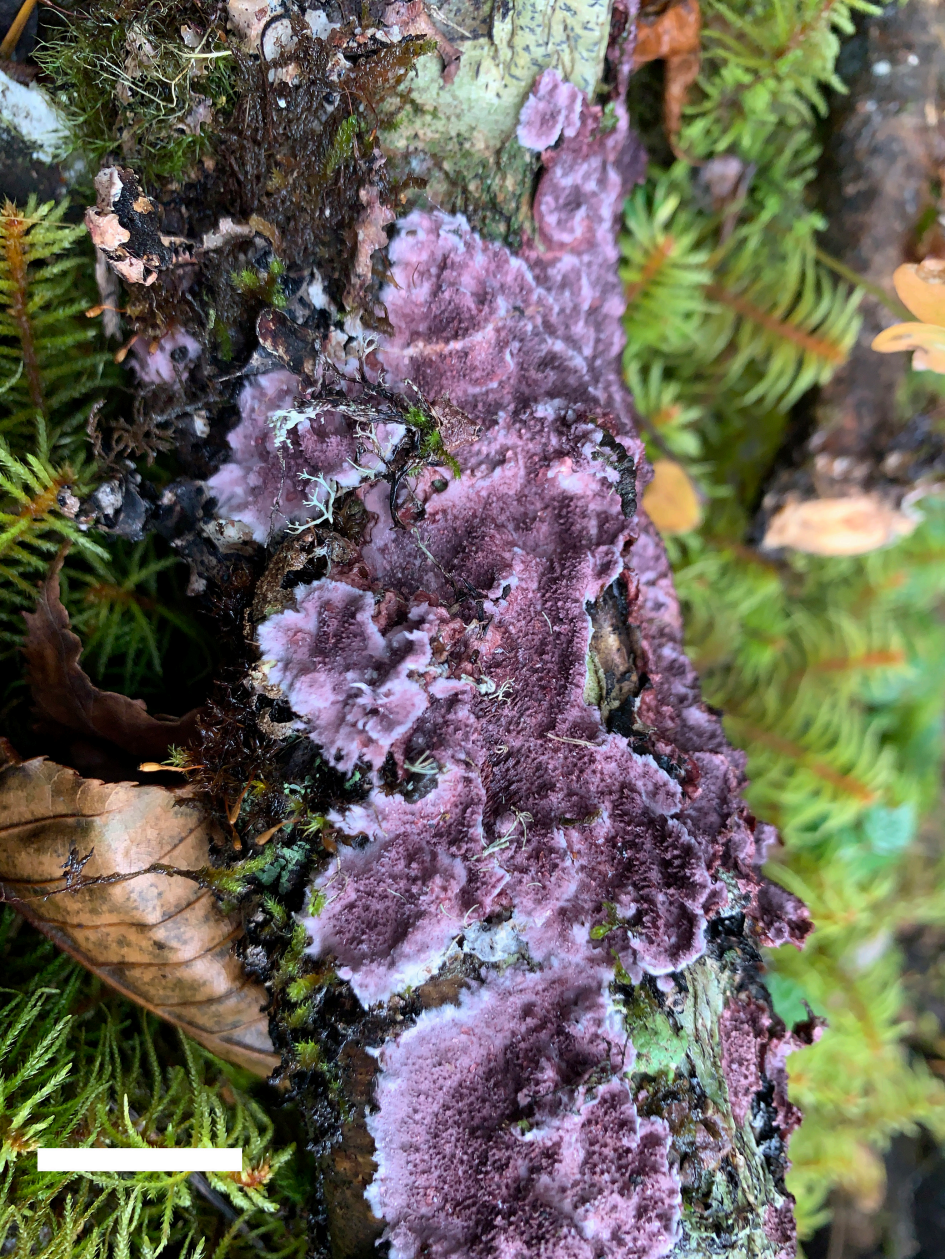
Basidiomata of *Etheirodon lilacinum* (holotype, Dai 23568). Scale bar: 1 cm.

**Figure 6.**
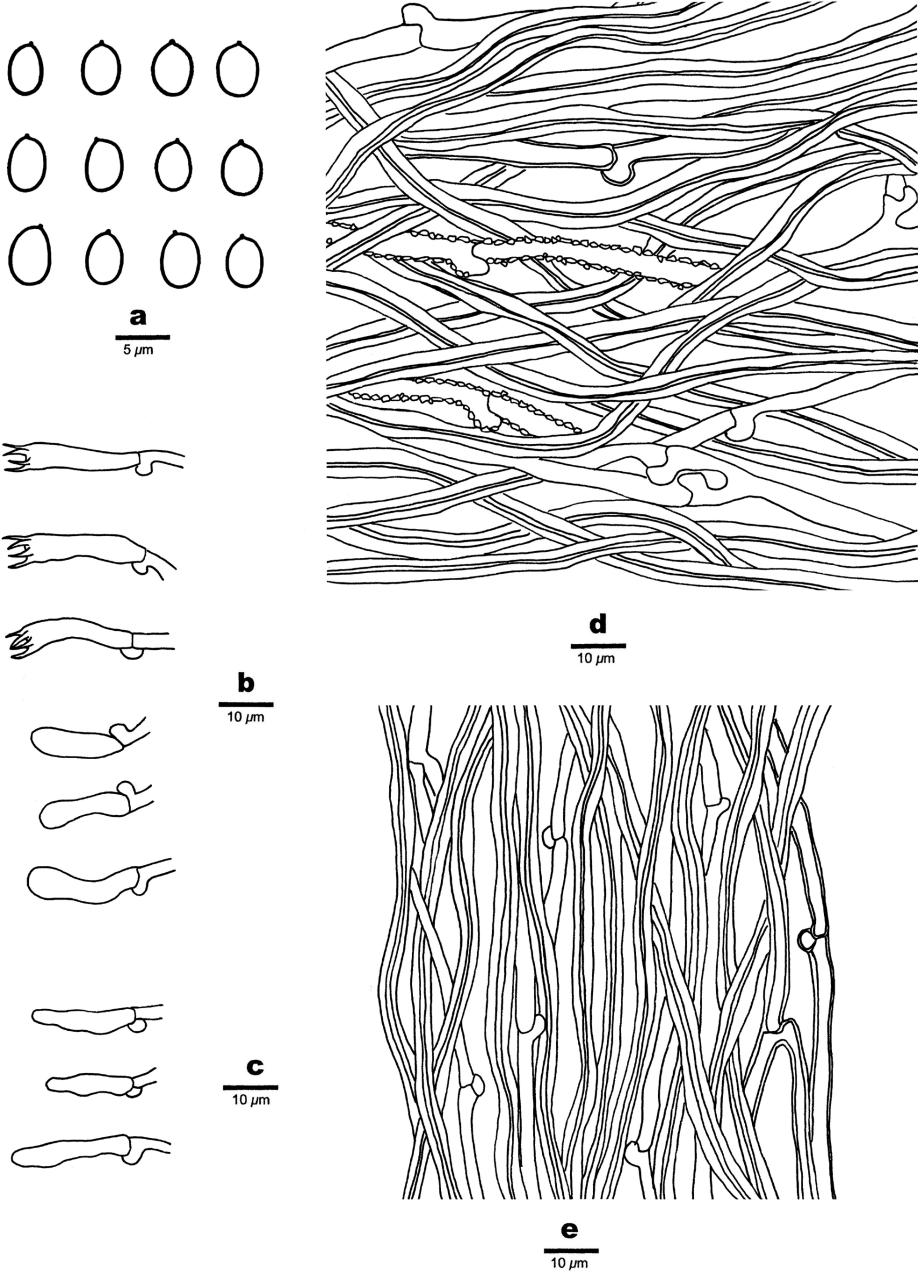
Microscopic structures of *Etheirodon lilacinum* (holotype, Dai 23568). **a**. Basidiospores; **b**. Basidia and basidioles; **c**. Cystidioles; **d**. Hyphae from context; **e**. Hyphae from spines.

##### Etymology.

*Lilacinum* (Lat.): refers to the species having lilac pore surface when fresh.

##### Description.

***Basidiomata***. Annual, resupinate to slightly effused-reflexed with small pilei, soft to fleshy, without odor or taste when fresh, soft corky when dry, up to 20 cm long, 3 cm wide when resupinate. Hymenophore hydnoid, lilac when fresh, dark grayish violet when dry; sterile margin distinct, lilac when fresh, grayish violet when dry, up to 3 mm wide, fimbriate to dentate; spines soft when fresh, soft corky when dry, up to 1 mm long, cylindrical to flattened, 2–4 per mm at base. Context pale grayish violet, soft corky when dry, up to 1.5 mm thick.

***Hyphal structure***. Hyphal system dimitic; generative hyphae bearing clamp connections; skeletal hyphae IKI−, CB−; tissues becoming pale olivaceous in KOH.

***Context***. Generative hyphae infrequent, hyaline, thin- to thick-walled with a wide lumen, sometimes covered with fine hyaline crystals, occasionally branched, straight, 3–4 μm in diam.; skeletal hyphae dominant, brownish orange, thick-walled with a narrow lumen to subsolid, smooth, rarely branched, more or less flexuous, loosely interwoven, 3–6 μm in diam.

***Spines***. Generative hyphae infrequent, hyaline, thin- to slightly thick-walled with a wide lumen, smooth, rarely branched, straight, 3–3.5 μm in diam.; skeletal hyphae dominant, brownish orange, thick-walled with a medium to narrow lumen, smooth, occasionally branched, straight, subparallel along the spines, agglutinated, 3.5–4 μm in diam. Cystidia absent; cystidioles fusoid, hyaline, thin-walled, smooth, 15–20 × 4 μm. Basidia clavate, with four sterigmata and a basal clamp connection, 20–23 × 4.5–5 μm; basidioles in shape similar to basidia, but smaller. Irregular crystals present amongst hymenia.

***Spores***. Basidiospores ellipsoid to broadly ellipsoid, hyaline, thin-walled, smooth, IKI−, CB−, (3.8–)4–4.5(–5) × (2.8–)3–3.8(–4) µm, L = 4.12 μm, W = 3.2 μm, Q = 1.27–1.3 (n = 90/3).

***Type of rot***. White rot.

##### Additional specimens examined.

China • Sichuan Province, Ganzi, Luding County, Hailuogou Forest Park, on fallen branch of *Abies*, 8 October 2021, Dai 23131 (BJFC037702), Dai 23140 (BJFC037711); Xizang Autonomous Region, Linzhi, Bomi County, on fallen trunk of *Betula*, 26 October 2021, Dai 23571 (BJFC038143), Dai 23574 (BJFC038146).

##### Notes.

*Etheirodon lilacinum* is characterized by resupinate to slightly effused-reflexed basidiomata; a hydnoid and lilac hymenophore when fresh, dark grayish violet when dry; cylindrical to flattened spines of 2–4 per mm at the base; a dimitic hyphal system bearing clamp connections on generative hyphae; thin- to thick-walled generative hyphae sometimes covered with fine hyaline crystals; ellipsoid to broadly ellipsoid basidiospores measuring 4–4.5 × 3–3.8 µm; and growing on angiosperm and gymnosperm wood.

### Key to genera within *Steccherinaceae*

**Table:** 

1	Hyphal system monomitic	**2**
–	Hyphal system dimitic or dimitic to trimitic	**10**
2	Hymenophore odontoid, tuberculate or smooth	*** Cabalodontia ***
–	Hymenophore poroid	**3**
3	Basidiomata resupinate	**4**
–	Basidiomata effused-reflexed, pileate to stipitate	**7**
4	Pore surface brown	*** Atraporiella ***
–	Pore surface light-colored	**5**
5	Ampullaceous septa and gloeocystidia present	*** Caudicicola ***
–	Ampullaceous septa and gloeocystidia absent	**6**
6	Tubes shallow, pores large and angular, sterile margin distinct	*** Niemelaea ***
–	Tubes long, pores small and round to angular, sterile margin absent	*** Bambusiporia ***
7	Hyphae in context swollen	*** Xanthoporus ***
–	Hyphae in context normal	**8**
8	Basidiospores thin-walled	*** Rhomboidia ***
–	Basidiospores thick-walled	**9**
9	Pilei usually imbricate with a common stipe, pores small	*** Flabellophora ***
–	Pilei usually solitary with a stipe or not, pores large	*** Loweomyces ***
10	Gloeocystidia present	**11**
–	Gloeocystidia absent	**13**
11	Basidiomata resupinate to pileate	*** Antrodiella ***
–	Basidiomata completely resupinate	**12**
12	Pore surface white to yellow	*** Antella ***
–	Pore surface straw-colored	*** Butyrea ***
13	Hymenophore completely poroid	**14**
–	Hymenophore poroid, odontoid, irpicoid, lamellae or corticioid	**19**
14	Pore surface sulfur-yellow or citric-yellow	**15**
–	Pore surface white, reddish, or brownish vinaceous	**16**
15	Basidiospores subcylindrical	*** Austeria ***
–	Basidiospores broadly ellipsoid to subglobose	*** Citripora ***
16	Encrusted thick-walled cystidia absent	**17**
–	Encrusted thick-walled cystidia present	**18**
17	Basidiospores allantoid	*** Nigroporus ***
–	Basidiospores oblong-ellipsoid	*** Frantisekia ***
18	Basidiomata resupinate	*** Junghuhnia ***
–	Basidiomata pileate	*** Flaviporus ***
19	Sterile margin fimbriate-rhizomorphic	*** Etheirodon ***
–	Sterile margin smooth	**20**
20	Encrusted thick-walled cystidia present	**21**
–	Encrusted thick-walled cystidia absent	**22**
21	Hyphal system trimitic, skeletal hyphae rather wide	*** Metuloidea ***
–	Hyphal system dimitic, skeletal hyphae narrow	*** Steccherinum ***
22	Basidiomata pileate without stipes	*** Lamelloporus ***
–	Basidiomata pileate with stipes	**23**
23	Basidiospores cylindrical and curved	*** Trullella ***
–	Basidiospores cylindrical to broadly ellipsoid	*** Mycorrhaphium ***

## Discussion

In the present study, phylogenetic analyses using a four-marker dataset (ITS + nLSU + *tef1* + mtSSU) illustrate the phylogeny of genera belonging to *Steccherinaceae* in *Polyporales* (Fig. 1). *Bambusiporia* forms an independent clade and is proposed as a new genus. Recently, divergence time estimation has been applied in fungal taxonomy, especially in genera, families, or higher-ranking taxa, to support fungal systematics (Chen et al. 2015; Varga et al. 2019; Zhao et al. 2023, 2025; Dong et al. 2024). In the present study, divergence time estimation of *Bambusiporia* was analyzed, and the result supports our proposal for the establishment of the new genus. Multi-gene phylogenetic analysis is a core approach for resolving phylogenetic relationships and delimiting taxonomic units in fungal systematics (Miettinen et al. 2012). Sufficient and matched sequence quantities across all markers can provide accurate resolution of phylogenetic relationships among closely related taxa. However, quantitative disparities among individual sequence fragments will lead to deviations in multi-gene analyses, and sampling more individuals often gives better results than sampling more loci in phylogenetic studies at the genus level (James et al. 2006; Maddison and Knowles 2006). In this study, a total of 77 samples representing 55 taxa were studied in our phylogenetic analysis, including 77 ITS sequences, 66 nLSU sequences, 16 mtSSU sequences, and 12 *tef1* sequences obtained. Phylogenetic results from multi-gene (ITS + nLSU + *tef1* + mtSSU) and two-gene (ITS + nLSU) analyses showed similar topology.

Bamboos are monocotyledonous woody plants. Fewer studies on bamboo-decaying fungi were reported before the 21^st^ century. However, recently the diversity of these fungi has been investigated, especially in China, and more than 20 new taxa of wood-decaying fungi on bamboos have been described (Dai et al. 2021; Wang et al. 2021; Mao et al. 2023; Zhang et al. 2023; Zhou et al. 2023; Cui et al. 2025). It seems that more unknown taxa of bamboo-decaying fungi will be described after further investigation. The present study proposes a new genus and a new species on bamboos from China.

*Bambusiporia* is a light-colored polypore that forms a separate, strongly supported clade in *Steccherinaceae* in our phylogenetic tree. So far, 24 genera accepted in *Steccherinaceae* have various types of hymenophores (poroid, hydnoid, and corticioid, Table 2). In the dating analyses (Fig. 2), the new genus *Bambusiporia* was estimated to have emerged in the late Cretaceous with a mean crown age of 83.23 Myr. However, the divergence time of the *Citripora* clade and the *Steccherinum* s.s. clade, with mean crown ages of 44.62 Myr and 52.09 Myr, respectively, both occurred during the Eocene in the Paleogene and are posterior to *Bambusiporia*. So far, the *Bambusiporia* clade diverged earlier than other genera in *Steccherinaceae* (Fig. 2). The divergence times of the *Poaceae* with a crown age of 101 Myr occurred during the early Cretaceous, and the ancestor of the bamboos (*Poaceae*: *Bambusoideae*) evolved during the Cretaceous–Paleogene (K–Pg) boundary at 66 Myr (Huang et al. 2022). In contrast, the new genus *Bambusiporia* was estimated to have diverged at 83.23 Myr in the late Cretaceous, predating the origin of *Bambusoideae*. Thus, *Bambusiporia* likely originally decomposed other monocotyledonous plants or gymnosperms and subsequently underwent a host shift to bamboo, a pattern consistent with that of some species such as *Meripilus cinereus* and *M. lineatus*, which are known to associate with both bamboos and gymnosperms as hosts (Wang et al. 2025).

**Table 2.** The main morphological characteristics of all 24 genera in *Steccherinaceae*.

Genera	Type species	Basidiomata	Type of hymenophore	Hyphal system	Shape of basidiospores	References
* Antella *	*A. niemelaei*	Resupinate	Poroid to irpicoid	Dimitic	Ellipsoid	Miettinen and Ryvarden (2016)
* Antrodiella *	*A. semisupina*	Resupinate to pileate	Poroid	Dimitic to trimitic	Cylindrical to ellipsoid	Ryvarden and Gilbertson (1993); Yuan (2014)
* Atraporiella *	*A. neotropica*	Resupinate	Poroid	Monomitic	Ellipsoid to slightly allantoid	Ryvarden (2007)
* Austeria *	*A. citrea*	Pileate	Poroid	Dimitic	Subcylindrical	Miettinen and Ryvarden (2016)
*** Bambusiporia ***	*B. nivea*	Resupinate	Poroid	Monomitic	Ellipsoid	This study
* Butyrea *	*B. luteoalba*	Resupinate	Poroid	Dimitic	Cylindrical	Miettinen and Ryvarden (2016)
* Cabalodontia *	*C. queletii*	Resupinate	Odontoid, tuberculate or smooth	Monomitic	Ellipsoid to subglobose	Piątek (2004)
* Caudicicola *	*C. gracilis*	Resupinate	Poroid	Monomitic	Broadly ellipsoid to subglobose	Kotiranta et al. (2017)
* Citripora *	*C. bannaensis*	Effused-reflexed to pileate	Poroid	Dimitic	Broadly ellipsoid to subglobose	Miettinen and Ryvarden (2016)
* Etheirodon *	*E. fimbriatus*	Resupinate to effused-reflexed	Odontoid to hydnoid	Dimitic	Ellipsoid to broadly ellipsoid	This study
* Flabellophora *	*F. superposita*	Pileate to stipitate	Poroid	Monomitic	Subglobose to globose	Cunningham (1965)
* Flaviporus *	*F. brownii*	Resupinate to effused-reflexed	Poroid	Dimitic	Ellipsoid, broadly ellipsoid to subglobose	Ryvarden and Gilbertson (1993); Wei et al. (2023)
* Frantisekia *	*F. fissiliformis*	Resupinate, effused-reflexed to pileate	Poroid	Dimitic	Oblong-ellipsoid	Spirin and Zmitrovich (2007); Yuan (2014)
* Junghuhnia *	*J. crustacea*	Resupinate to rarely effused-reflexed	Poroid	Dimitic	Cylindrical to ellipsoid	Ryvarden and Gilbertson (1993)
* Lamelloporus *	*L. americanus*	Pileate	Lamellae	Dimitic	Ellipsoid	Ryvarden (1987)
* Loweomyces *	*L. fractipes*	Resupinate, effused-reflexed to stipitate	Poroid	Monomitic	Broadly ellipsoid to subglobose	Kotlába and Pouzar (1976)
* Metuloidea *	*M. tawa*	Effused-reflexed to pileate	Poroid to hydnoid	Dimitic to trimitic	Ellipsoid to short cylindrical	Cunningham (1965)
* Mycorrhaphium *	*M. adustum*	Effused-reflexed, pileate to stipitate	Poroid to hydnoid	Dimitic	Cylindrical to ellipsoid	Maas Geesteranus (1971); Westphalen et al. (2019)
* Niemelaea *	*N. consobrina*	Resupinate	Poroid	Monomitic	Ellipsoid to broadly ellipsoid	Ryvarden and Gilbertson (1993); Zmitrovich et al. (2015)
* Nigroporus *	*N. vinosus*	Resupinate to pileate	Poroid	Dimitic	Allantoid	Ryvarden and Johansen (1980)
* Rhomboidia *	*R. wuliangshanensis*	Pileate	Poroid	Monomitic	Broadly ellipsoid	Xu et al. (2020)
* Steccherinum *	*S. ochraceum*	Resupinate, effused-reflexed to pileate or stipitate	Poroid, odontoid to corticioid	Dimitic	Ellipsoid to subcylindrical	Liu et al. (2023)
* Trullella *	*T. dentipora*	Pileate to stipitate	Poroid to irpicoid	Dimitic	Cylindrical and curved	Zmitrovich (2018)
* Xanthoporus *	*X. peckianus*	Pileate to stipitate	Poroid	Monomitic	Ellipsoid to subglobose	Audet (2010)

*Citripora* encompasses the two species, *C. afrocitrina* (Ipulet & Ryvarden) Miettinen & Ryvarden and *C. bannaensis* Miettinen. It has effused-reflexed to pileate basidiomata with lemon-yellow tints, small pores, a dimitic hyphal system bearing clamp connections on generative hyphae, and tiny, broadly ellipsoid to subglobose basidiospores (Miettinen and Ryvarden 2016). Though *Citripora* grouped with *Bambusiporia* in a joint clade, the divergence time of them differs by nearly 40 Myr (Fig. 2). In addition, the *Citripora* clade is phylogenetically close to the *Bambusiporia* clade without strong support (Fig. 1). Polypore genera *Flabellophora*, *Nigroporus*, *Rhomboidia*, and *Trullella* have effused-reflexed to pileate or stipitate basidiomata (Cunningham 1965; Ryvarden and Johansen 1980; Miettinen and Ryvarden 2016; Xu et al. 2020), which differs from *Bambusiporia*. In addition, *Flabellophora* and *Rhomboidia* have broadly ellipsoid to subglobose basidiospores; *Nigroporus* has grayish-blue, vinaceous-brown to dark brown basidiomata, a dimitic hyphal system, and allantoid basidiospores; *Trullella* has a dimitic hyphal system and allantoid basidiospores, and these characteristics are also different from *Bambusiporia*. *Mycorrhaphium* is a pileate hydnoid genus that encompasses eleven species. *Austeria* is monotypic and is characterized by pileate basidiomata with sulfur-yellow tints, tiny pores, a dimitic hyphal system, and subcylindrical and thin-walled basidiospores (Miettinen and Ryvarden 2016), while *Bambusiporia* is a resupinate poroid genus.

*Niemelaea* features three species, viz., *N. balaenae* (Niemelä) V. Papp, *N. consobrina* (Bres.) Zmitr. et al., and *N. cremea* (Parmasto) Zmitr., Ezhov & Khimich. It has resupinate basidiomata with a light-colored pore surface, a monomitic hyphal system bearing clamp connections on generative hyphae, and ellipsoid to broadly ellipsoid basidiospores (Zmitrovich et al. 2015). These characteristics are similar to *Bambusiporia*. However, *Niemelaea* is unrelated to *Bambusiporia* in the phylogeny (Fig. 1).

*Etheirodon fimbriatus* (basionym: *Odontia fimbriata* Pers.) was originally described from France. It has pinkish basidiomata; an odontioid hymenophore, usually with a filamentous to rhizomorphic sterile margin; conical spines of 4–5 per mm at the base; strongly encrusted and thick-walled cystidia; and ellipsoid basidiospores (Eriksson et al. 1984). *Etheirodon purpureus* was originally described from Brazil. It is very similar to *E. fimbriatus*, but the former has smaller spines of 7–10 per mm at the base (Westphalen et al. 2021). *Etheirodon fimbriatus* and *E. purpureus* differ from the new species *E. lilacinum* by having an ochraceous to dark ochraceous hymenophore when dry, the presence of encrusted and thick-walled cystidia, and smaller basidiospores (3.5–4 × 2–2.5 µm in *E. fimbriatus* and 4–4.5 × 2–2.5 µm in *E. purpureus* vs. 4–4.5 × 3–3.8 µm in *E. lilacinum*, Westphalen et al. 2021). The two specimens, Dai 24450 and CLZhao 13977, were found in China, and their morphological characteristics are very similar to *E. fimbriatus*. The phylogenetic analysis suggests a close relatedness to *E. fimbriatus*. In this study, we treat them as *Etheirodon cf. fimbriatus*. In addition, specimens KUC 20121109-29 and HHB-2878-sp are treated as “*Etheirodon cf. fimbriatus*” as well because we did not study their voucher material. *Etheirodon* diverged in the Miocene with a mean crown age of 19.56 Myr. *Etheirodon lilacinum* diverged from *E. fimbriatus* s.l. during the late Miocene with a mean crown age of 6.03 Myr.

## Supplementary Material

XML Treatment for Bambusiporia

XML Treatment for Bambusiporia nivea

XML Treatment for Etheirodon lilacinum

## Additional information

### Use of AI

No use of AI was reported.

### Author ORCIDs

Chao-Ge Wang https://orcid.org/0000-0003-4381-5720

Xin Zhang https://orcid.org/0009-0005-8363-7852

Zhan-Bo Liu https://orcid.org/0000-0002-3894-5398

Jian Chen https://orcid.org/0000-0001-8409-7791

Yi-Fei Sun https://orcid.org/0000-0003-3997-3662

Yu-Cheng Dai https://orcid.org/0000-0002-6523-0320

Ying-Da Wu https://orcid.org/0000-0003-1295-4015

### Data availability

All of the data that support the findings of this study are available in the main text or Supplementary Information.

## Supplementary materials

Supplementary material 1 Phylogenetic analyses Data type rar https://binary.pensoft.net/file/1565761This dataset is made available under the Open Database License (http://opendatacommons.org/licenses/odbl/1.0/). The Open Database License (ODbL) is a license agreement intended to allow users to freely share, modify, and use this Dataset while maintaining this same freedom for others, provided that the original source and author(s) are credited. Chao-Ge Wang, Xin Zhang, Zhan-Bo Liu, Jian Chen, Yi-Fei Sun, Yu-Cheng Dai, Ying-Da Wu
